# Comment on “When the Outcome Contains the Exposure: Methodological Limits of a Genome‐Wide Cross‐Trait Analysis of Type 2 Diabetes and MASLD”

**DOI:** 10.1155/humu/4135090

**Published:** 2026-06-25

**Authors:** Qingjie Wu, Cai Zhang

**Affiliations:** ^1^ Research Base of Traditional Chinese Medicine Syndrome, Fujian University of Traditional Chinese Medicine, Fuzhou, China, fjtcm.edu.cn; ^2^ Department of Health and Kinesiology, University of Illinois Urbana-Champaign, Urbana, Illinois, USA, illinois.edu; ^3^ Beckman Institute for Advanced Science and Technology, University of Illinois Urbana-Champaign, Urbana, Illinois, USA, illinois.edu

We commend Zhu et al. [1] for a thorough cross‐trait pipeline relating type 2 diabetes mellitus (T2DM) to metabolic dysfunction–associated steatotic liver disease (MASLD), but three internal‐consistency problems undercut the headline causal and shared‐architecture claims.

First, the headline link is partly built into the outcome. The 2023 multisociety nomenclature defines MASLD as hepatic steatosis plus at least one cardiometabolic criterion, and T2DM (or hyperglycemia) is explicitly one such criterion [2], so reading a T2DM‐to‐MASLD effect as a discovery is partly tautological regardless of the GWAS used; the authors compound this by treating their NAFLD‐to‐MASLD relabeling as reinforcing the result. Separately, at the data level, the proxy NAFLD GWAS comprises 8434 cases against 770,180 controls under an assumed population prevalence of 32.4% [3], which implies heavy control contamination by undiagnosed steatosis and biases the genetic correlation and Mendelian randomization (MR) toward the null.

Second, the flagship forward MR fails its own pleiotropy test. The primary estimate (OR = 1.118) carries a significant MR‐Egger intercept (*p* = 7.40 × 10^−4^), a direct signal of directional horizontal pleiotropy [4], and the effect collapses to OR = 1.064 (*p* = 0.037) once the authors tighten their threshold to de‐pleiotropize. The nonsignificant MR‐PRESSO global test and Cochran′s *Q* (both near *p* = 0.90) do not rescue a significant Egger intercept and can reflect limited power to detect outliers.

Third, “no reverse causality” is a power artifact. The forward direction rests on 242,283 cases versus 8434 for the reverse, so a null reverse estimate cannot be read as evidence of absence [5], and the authors′ own discussion concedes that “the possibility of MASLD promoting T2DM remains plausible,” which sits uneasily beside the abstract′s categorical claim.

Several secondary issues reinforce the pattern. The global genetic correlation rests on a sample‐overlap assumption the paper contradicts internally, because Section 2.3 states there is no overlap and fixes the constrained intercept that yields *r*_g_ = 0.80, whereas Sections 2.8 and 3.5 acknowledge overlap and apply MRlap [6]. Two of the highly shared variants are canonical direct hepatic‐fat effectors, PNPLA3 (rs738408) and TM6SF2 (rs58542926), so a shared signal conflates horizontal pleiotropy with shared causal architecture [7, 8]. And the five genes highlighted at 19p13.11 lie within one correlated linkage‐disequilibrium block, with the original study [1] using nearest‐gene labels (rs10404726 maps to CRTC1, and rs7203132 is intergenic), so this is a locus‐level signal, not five independent causal targets.

These claims would be substantiated by reporting per‐direction instrument strength and power, presenting overlap‐robust estimates as primary, a sensitivity analysis excluding known hepatic‐lipid variants, fine‐mapping before any locus‐level target claim, and STROBE‐MR‐compliant reporting with data accessions and code [9]. The underlying question is important, and these refinements would calibrate the conclusions to what the data support.

## Funding

No funding was received for this manuscript.

## Conflicts of Interest

The authors declare no conflicts of interest.

## Data Availability

No new data were generated or analyzed in support of this Letter; it discusses publicly available GWAS summary statistics cited herein.
